# Use of Cell Viability Assay Data Improves the Prediction Accuracy of Conventional Quantitative Structure–Activity Relationship Models of Animal Carcinogenicity

**DOI:** 10.1289/ehp.10573

**Published:** 2008-01-04

**Authors:** Hao Zhu, Ivan Rusyn, Ann Richard, Alexander Tropsha

**Affiliations:** 1 Carolina Environmental Bioinformatics Research Center; 2 Laboratory for Molecular Modeling, Division of Medicinal Chemistry and Natural Products, School of Pharmacy and; 3 Department of Environmental Sciences and Engineering, School of Public Health, University of North Carolina at Chapel Hill, Chapel Hill, North Carolina USA; 4 National Center for Computational Toxicology, Office of Research and Development, U.S. Environmental Protection Agency, Research Triangle Park, North Carolina, USA

**Keywords:** carcinogenesis, computational toxicology, high-throughput screening, QSAR

## Abstract

**Background:**

To develop efficient approaches for rapid evaluation of chemical toxicity and human health risk of environmental compounds, the National Toxicology Program (NTP) in collaboration with the National Center for Chemical Genomics has initiated a project on high-throughput screening (HTS) of environmental chemicals. The first HTS results for a set of 1,408 compounds tested for their effects on cell viability in six different cell lines have recently become available via PubChem.

**Objectives:**

We have explored these data in terms of their utility for predicting adverse health effects of the environmental agents.

**Methods and results:**

Initially, the classification *k* nearest neighbor (*k*NN) quantitative structure–activity relationship (QSAR) modeling method was applied to the HTS data only, for a curated data set of 384 compounds. The resulting models had prediction accuracies for training, test (containing 275 compounds together), and external validation (109 compounds) sets as high as 89%, 71%, and 74%, respectively. We then asked if HTS results could be of value in predicting rodent carcinogenicity. We identified 383 compounds for which data were available from both the Berkeley Carcinogenic Potency Database and NTP–HTS studies. We found that compounds classified by HTS as “actives” in at least one cell line were likely to be rodent carcinogens (sensitivity 77%); however, HTS “inactives” were far less informative (specificity 46%). Using chemical descriptors only, *k*NN QSAR modeling resulted in 62.3% prediction accuracy for rodent carcinogenicity applied to this data set. Importantly, the prediction accuracy of the model was significantly improved (72.7%) when chemical descriptors were augmented by HTS data, which were regarded as biological descriptors.

**Conclusions:**

Our studies suggest that combining NTP–HTS profiles with conventional chemical descriptors could considerably improve the predictive power of computational approaches in toxicology.

The traditional approaches for *in vivo* animal chemical safety testing are costly, time consuming, and have a low throughput (Bucher and Portier 2004). To improve the efficiency of assessing potential human health hazards of environmental chemicals, the National Toxicology Program (NTP) at the National Institute of Environmental Health Sciences (NIEHS) recently initiated the High Throughput Screening (HTS) project (NTP 2007; Inglese et al. 2006; Xia et al. 2007). The NTP–HTS effort aims to develop high-throughput biological assays that aid in predicting a chemical’s potential for *in vivo* toxicity in a manner that is both informative of mechanisms and pathways and relevant to human health risk assessment. These assays are expected to help in prioritizing compounds for targeted animal testing. Recently, a set of 1,408 chemical agents, many with known *in vivo* toxicity profiles, was screened in six human cell lines for cytotoxicity and other phenotypic end points. The HTS results, including complete dose–response data for all tested compounds, were made publicly available through PubChem [National Center for Biotechnology Information (NCBI) 2007]. These data can be explored in terms of assessing the relevance of HTS screening to predictive toxicology.

Accurate prediction of the adverse effects of chemical substances on living systems, identification of possible toxic alerts, and compound prioritization for animal testing are the primary goals of computational toxicology. Rapid expansion of experimental data sets that combine data on chemical structure and various toxicity end points for numerous environmental agents {e.g., NTP [NTP 2007]; Berkeley Carcinogenic Potency Database [CPDB 2007]; and Distributed Structure-Searchable Toxicity database [DSSTox; U.S. Environmental Protection Agency (U.S. EPA) 2007]} provides novel opportunities to explore the relationships between chemical structure and toxicity using cheminformatics approaches. Application of advanced cheminformatics tools, such as quantitative structure–activity relationship (QSAR) methods, to the analysis of these data may provide means for accurate prediction of chemical toxicity of untested compounds, allowing for prioritization of compounds for subsequent animal testing.

QSAR modeling aims to establish rigorous correlations between the chemical descriptors of a set of compounds and their experimentally studied biological activities. Many different QSAR approaches have been developed over nearly 50 years of research (Beresford et al. 2004; Dearden 2003; Johnson et al. 2004; Schultz et al. 2003a). Recent trends in the field have focused on model validation as the key part of model development to ensure significant external predictive power of QSAR models. Traditional QSAR models are developed based on chemical descriptors alone (Klopman et al. 2004; Richard 2006). In some cases, additional physicochemical properties, such as water partition coefficient (logP) (Klopman et al. 2003), water solubility (Stoner et al. 2004), and melting point (Mayer and Reichenberg 2006) were used successfully to augment computed chemical descriptors and improve the predictive power of QSAR models. These studies suggest that using hybrid descriptor sets in QSAR modeling could prove beneficial.

The availability of HTS data on large sets of chemical agents offers an attractive avenue for exploring its utility in hybrid descriptor-based QSAR modeling. In this respect, the NTP–HTS data represent attractive and potentially mechanistically relevant *in vitro* “biological” descriptors for modeling the adverse health effects *in vivo*. Our study tested a hypothesis that improved QSAR predictions can be developed using a combination of chemical and biological descriptors of environmental chemicals. To this end, we have developed QSAR models based on NTP–HTS data using the *k* nearest neighbor (*k*nn) approach. Initially, we modeled the NTP–HTS results separately to explore the inherent relationship between chemical structure and its effect on cell viability. Next, we evaluated if a correlation exists between the NTP–HTS assay results and their *in vivo* rodent carcinogenic potency, as reported in the CPDB. Subsequently, the HTS results were used as biological descriptors that were combined with chemical descriptors to develop *k*NN QSAR models for predicting rodent carcinogenicity summary calls of the compounds. Finally, we attempted to examine the relative significance of the HTS “descriptors” in the resulting models and their interplay with chemical descriptors. Our studies demonstrate that adding NTP–HTS data to chemical descriptors employed in conventional QSAR modeling affords improved models that may advance the use of computational approaches in toxicology.

Our current studies were limited to exploring the value of cell viability assays in predicting rodent carcinogenicity as one example of *in vivo* toxicity end point. This limitation was because *in vivo* rodent carcinogenicity is the only end point reported in the CPDB for a significant fraction of compounds also tested for their effect on cell viability. Certainly, as additional chemicals with known *in vivo* responses are tested in cell-based assays, we will continue to explore similar approaches in correlating the *in vitro* and *in vivo* data.

## Methods

### Data sources

#### NTP–HTS data set

The NTP–HTS assay results were obtained from PubChem (NCBI 2007), and chemical structures associated with these results were provided by the DSSTox (U.S. EPA 2007) database. The complete data set included 1,408 compounds that were tested in six cell lines at the National Institutes of Health (NIH) Chemical Center Genomics (NCGC) (Inglese et al. 2006; Xia et al. 2007). The cell lines used for screening of the effect of chemical agents on cell viability included BJ [human foreskin fibroblast; PubChem bioassay identifier (AID) no. 421], HEK293 (transformed human embryonic kidney cell line; AID no. 427), HepG2 (human hepatoma; AID no. 433), Jurkat (clone E6-1, human acute T-cell leukemia; AID no. 426), MRC-5 (human lung fibroblast; AID no. 434), and SK-N-SH (human neuroblastoma; AID no. 435). Details on the assays and the testing protocols can be found in PubChem. For the purposes of this work, the data set was curated as follows. First, we removed duplicate data entries for 55 chemical records with identical chemical structures (i.e., keeping one of the two identical records) and 14 records for which molecular structure could not be obtained. Second, inorganic and organometallic compounds as well as compound mixtures were excluded since these do not have conventional chemical descriptors used in QSAR studies. The curated subset of the original NTP–HTS data set used in this work included 1,289 unique organic compounds [Supplemental Material, Table 1 (online at http://www.ehponline.org/members/2008/10573/suppl.pdf)]. The “activity” classification for each compound, for each HTS assay, was assigned by NCGC as reported in PubChem. HTS studies included the 55 duplicate compounds. The analysis of assay results for these duplicate compounds demonstrated that the HTS data were highly reproducible (Xia et al. 2007).

#### The CPDB database

We obtained the rodent carcinogenicity data from the CPDB (CPDB 2007; Gold et al. 1991). The CPDB provides a systematic and unifying source of the outcomes from *in vivo* animal chemical carcinogenicity studies. The most recent release of the CPDB includes experimental data on testing of 1,481 diverse chemicals in one or both sexes of rats and mice, reporting outcomes on 35 possible target organ/tissue sites. A chemical structure–annotated version of the CPDB summary tables consolidating all species was published on the U.S. EPA DSSTox website (U.S. EPA 2007) with additional summary activity categorizations and was used for the present study. For modeling purposes, chemical agents in the CPDB were categorized as follows: active (multisite, multi-sex, or multispecies carcinogens), marginally active (single-site carcinogens), inactive (non-carcinogenic in more than two test cells and no active results), or no conclusion (insufficient results). Of the 1,466 compounds classified as “active” or “inactive” in the CPDB, 314 were represented in the NTP–HTS data set (178 active and 136 inactive) and used in this study. A complete list of these agents is provided in the Supplemental Material, Table 2 (online at http://www.ehponline.org/members/2008/10573/suppl.pdf).

### MolConnZ chemical descriptors

The MolConnZ software (eduSoft LC, Ashland, VA, USA) affords computation of a wide range of topologic indices of molecular structure. These indices include but are not limited to the following descriptors: simple and valence path, cluster, path/cluster and chain molecular connectivity indices, kappa molecular shape indices, topologic and electrotopologic state indices, differential connectivity indices, graph radius and diameter, Wiener and Platt indices, Shannon and Bonchev–Trinajstiç information indices, counts of different vertices, counts of paths, and edges between different kinds of vertices (Hall et al. 1991; Kier 1986, 1987; Kier and Hall 1991). Overall, MolConnZ produces over 400 different descriptors. Those with zero value or zero variance were removed. The remaining descriptors were range scaled, as the absolute scales for MolConnZ descriptors can differ by orders of magnitude. Accordingly, our use of range scaling avoided giving descriptors with significantly higher ranges a disproportional weight on distance calculations in multidimensional MolConnZ descriptor space.

### QSAR modeling

#### Selection of test and training sets

The curated NTP–HTS data set (consisting of 1,289 unique organic compounds) was subdivided into multiple training/test set pairs using the sphere exclusion program developed in our laboratory (Golbraikh et al. 2003). The number of compounds included in the test set was gradually increased to obtain the largest possible test set for which accurate predictions could be obtained from models developed for the corresponding smallest possible training set.

The procedure implemented in the present study begins with the calculation of the distance matrix *D* between points that represent compounds in the descriptor space. Let *D*_min_ and *D*_max_ be the minimum and maximum elements of *D*, respectively. *N* probe sphere radii, *R*, are defined by the following formulas: *R*_min_ = *R*_1_ = *D*_min_, *R*_max_ = *R_N_* = *D*_max_/4, *R_i_* = *R*_1_ + (*i*–1)*(*R_N_*–*R*_1_)/(*N*–1), where *i* = 2, ..., *N*–1. Each probe sphere radius corresponds to one division in the training and the test set. A sphere-exclusion algorithm used in the present study consisted of the following steps: (i) randomly select a compound; (ii) include it in the training set; (iii) construct a probe sphere around this compound; (iv) select compounds from this sphere and include them alternately into the test and the training sets; (v) exclude all compounds from within this sphere from further consideration; and (vi) if no more compounds are left, stop. Otherwise let *m* be the number of probe spheres constructed and *n* be the number of remaining compounds. Let *d_ij_* (*i*=1,...,*m; j*=1,...,*n*) be the distances between the remaining compounds and the probe sphere centers. Select a compound corresponding to the lowest *d_ij_* value and go to step (ii). This algorithm guarantees that at least in the entire descriptor space (i) representative points of the test set are close to representative points of the training set (test set compounds are within the applicability domain defined by the training set); (ii) most of the representative points of the training set are close to representative points of the test set; and (iii) the training set represents the entire modeling set (i.e., there is no subset in the modeling set that is not represented by a similar compound in the training set) (Golbraikh et al. 2003). Consequently, the sphere exclusion algorithm could maximize the diversity of the training/test sets in the descriptor space used for modeling. Because of the stochastic nature of the algorithm, the composition of training and test sets is different for different original data set divisions. For example, we tested the results of more than 40 data set divisions generated by the sphere exclusion and found that any two training sets had no more than 85% identical compounds.

The statistical significance of models was characterized with the standard leave-one-out cross-validated *R*^2^ (*q^2^*) for the training sets and the conventional *R^2^* for the test sets. Models were considered acceptable if both *q^2^* and *R^2^* were larger than the arbitrary cutoff values (0.65 was used as a cutoff in this study). Models that did not meet these cutoff criteria were discarded. Additional details of this approach are described elsewhere (Golbraikh et al. 2003; Golbraikh and Tropsha 2002b).

#### *k*NN QSAR method

The *k*NN QSAR method employs the *k*NN pattern recognition principle and a variable selection procedure. Initially, a subset of *nvar* (number of selected variables) descriptors is selected randomly. The model developed with this set of descriptors is validated by leave-one-out cross-validation, where each compound is eliminated from the training set, and its biological activity is predicted as the average activity of *k* most similar molecules (*k* = 1 to 5). The weighted molecular similarity was characterized by the modified Euclidean distance between compounds in the *nvar* subspace of the multidimensional descriptor space. Generally, the Euclidean distances in the descriptor space between a compound and each of its *k* nearest neighbors are not the same. Thus, the activity of each of the *k* neighbors of a compound was given a weight that was higher for close neighbors and lower for distant neighbors as follows (Equations 1 and 2):


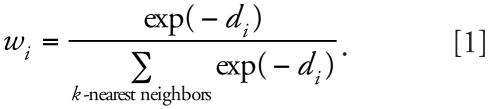



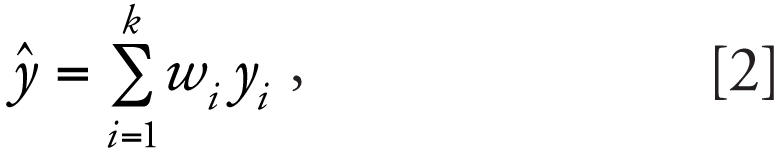


where *d_i_* is the Euclidean distance between the compound and its *k* nearest neighbors; *w_i_* is the weight for every individual nearest neighbor; *y_i_* is the actual activity value for nearest neighbor *i*; and *ŷ* is the predicted activity value. A method of simulated annealing with the Metropolis-like acceptance criteria is used to optimize the variable selection.

In summary, the *k*NN QSAR algorithm generates both an optimum *k* value and an optimal *nvar* subset of descriptors, that afford a QSAR model with the highest training set model accuracy as estimated by the *q^2^* value. Further details of the *k*NN method implementation, including the description of the simulated annealing procedure used for stochastic sampling of the descriptor space, are given in our previous publications (Ng et al. 2004; Shen et al. 2003; Zheng and Tropsha 2000).

#### Applicability domain of *k*NN QSAR models

Formally, a QSAR model can predict the target property for any compound for which chemical descriptors can be calculated. However, because all the models are developed in *k*NN QSAR modeling by interpolating activities of the nearest neighbor compounds only in the relevant training sets, a special applicability domain (i.e., similarity threshold) should be introduced to avoid making predictions for compounds that differ substantially from the training set molecules. This procedure resembles that for identifying chemical outliers prior to the onset of modeling.

To measure similarity, each compound is represented by a point in the *M*-dimensional descriptor space (where *M* is the total number of descriptors in the descriptor pharmacophore) with the coordinates *X_i_*_1_, *X_i_*_2_, ..., *X_iM_*, where *X_i_*’s are the values of individual descriptors. The molecular similarity between any two molecules is characterized by the Euclidean distance between their representative points. The Euclidean distance *d_i_*_,_*_j_* between two points *i* and *j* (which correspond to compounds *i* and *j)* in *M*-dimensional space can be calculated as follows (Equation 3):


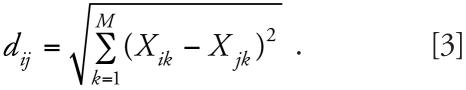


Compounds with the smallest distance between one another are considered to have the highest similarity. The similarities of compounds in our training set are compiled to produce an applicability domain threshold, *D_T_*, calculated as follows (Equation 4):


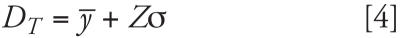


Here, *ȳ* is the mean Euclidean distance to the nearest neighbor of each compound within the modeling set, σ is the standard deviation of these Euclidean distances, and *Z* is an arbitrary parameter to control the significance level. On the basis of previous studies (Shen et al. 2002), we set the default value of this parameter to 0.5, which formally places the boundary for which compounds will be predicted at one-half of the SD (assuming a Boltzmann distance distribution between *k*NN compounds in the training set). Thus, if the distance of the external compound from at least one of its *k* nearest neighbors in the training set exceeds this threshold, the prediction is considered unreliable.

#### Robustness of QSAR models

y-Randomization (randomization of response) is a widely used approach to establish the model robustness. It consists of rebuilding the models using randomized activities of the modeling set and subsequent assessment of the model statistics. It is expected that models obtained for the modeling set with randomized activities should have significantly lower predictivity for the external validation set than the models built using the modeling set with real activities, or the total number of acceptable models based on the randomized modeling set satisfying the same cutoff criterion (*q^2^* and *R^2^* > 0.65) is much less than that based on the real modeling set. If this condition is not satisfied, real models built for this modeling set are not reliable and should be discarded. This test was applied to all data divisions considered in this study.

## Results

Table 1 provides a summary of the classification of the chemical agents used for these studies with respect to their “biological activity” (i.e., the effect on cell viability) in each of the six cell lines used for screening. In the entire NTP–HTS data set of unique 1,289 compounds, 140 were defined as “active” and 90 as “inconclusive” based on one or more active or inconclusive calls recorded in PubChem across the six cell lines, respectively. The majority of compounds—1,059—were recorded in PubChem as “inactive” in all experiments. Overall, the NTP–HTS data set contains 314 compounds that can be mapped to the CPDB database and classified as carcinogenic according to DSSTox “multisite, multisex, or multispecies” summary designations (Table 2).

### QSAR modeling of NTP–HTS data using chemical descriptors

QSAR modeling of the NTP–HTS data was desired to establish predictive models of HTS assays that can be used to impute such data for future compound libraries that may be tested. In addition, our use of the y-randomization test as part of modeling procedures could be viewed as an independent statistical test of the “nonrandomness” of the HTS data. The curated NTP–HTS data set has a biased distribution of active and inactive compounds (16% actives and inconclusives vs. 84% inactives). This is characteristic of most of the available biological data sets (such as those deposited in PubChem), which are dominated by inactive compounds. To address this bias, we used a (dis)similarity search to exclude a considerable fraction of inactive compounds from the data set to balance the active/inactive ratio for modeling purposes. To this end, we calculated the Molecular ACCess System (MACCS) structural keys (Renner and Schneider 2006) for all 1,289 compounds in the data set, using the MOE software (Chemical Computing Group, Montreal, Canada). All the active compounds were used as a probe subset, and the Tanimoto coefficients (Schultz et al. 2003b; Willett and Winterman 1986) between each inactive compound and the probe subset were calculated based on their MACCS keys. The inactive compound was selected into the modeling set only if it had a relatively high Tanimoto similarity (> 0.7) with one or more active/inconclusive compounds. Using this approach, 244 of the original 1,079 inactive compounds were selected because of their relatively high similarity to the active compounds. Thus, the final data set for the classification QSAR modeling included a total of 384 compounds (140 actives and 244 inactives). The rationale for this approach to selecting (a subset of) inactive compounds for the classification modeling is that it is more challenging to establish robust models when the two classes of active and inactive compounds include relatively similar molecules. It is quite obvious that if the two classes of compounds (i.e., active or inactive) are chemically dissimilar as judged by a simple similarity metric such as Tanimoto coefficients, then no additional statistical modeling using sophisticated data mining techniques is necessary. We did not include any compounds with inconclusive results in our modeling studies.

Because it is critical to demonstrate that QSAR models have high prediction accuracy for external validation data sets (Golbraikh and Tropsha 2002a; Zhang et al. 2006), 109 compounds (37 actives and 72 inactives) were randomly selected for external model validation. The remaining 275 compounds (103 actives and 172 inactives) were used for modeling, and multiple training and test sets were generated. The variable selection *k*NN QSAR models were developed for each training set, and the predictive power of each model was assessed against the corresponding test set. The acceptability cutoff values of the leave-one-out cross-validation accuracy and the prediction accuracy for the test set were set to 0.65 (Kovatcheva et al. 2004). Because the data set was unbalanced, we used the average of sensitivity and specificity to represent the overall predictive power of a model in this study. Therefore, the overall predictive accuracy of each model was defined as the average of the correctly predicted active ratio (sensitivity) and the correctly predicted inactive ratio (specificity) (de Lima et al. 2006). The total number of models that satisfied the accuracy threshold criteria was 599, and the statistical characteristics of 15 most significant *k*NN models are shown in Table 3.

Our previous studies have demonstrated that the highest external prediction accuracy of QSAR models is achieved using the consensus approach, that is, by averaging the predictions from individual models (Tropsha et al. 2003). The consensus prediction results for 109 compounds in the external validation set are provided in Table 4. The sensitivity and specificity of the consensus prediction were 56.8% and 90.2%, respectively. Thus, the overall predictive power was 73.5%, that is, similar to that for the training/test sets (Table 3).

To ensure high external validation accuracy of the training set models, we also considered their applicability domains. This restriction decreases the number of compounds considered for the prediction but increases the reliability so that higher accuracy is typically expected. Indeed, after removing compounds outside the applicability domain of our training set models, the coverage of the external set was reduced to 88%. However, the accuracy of prediction for actives and inactives improved to 65.4% and 92.9%, respectively (i.e., total accuracy increased to ~ 80%).

It is interesting to see whether the *k*NN HTS models could make reliable predictions of the remaining 835 inactive compounds, which were excluded because they were relatively dissimilar to the compounds used in the modeling procedure. The consensus prediction gave 64.1% predictive accuracy for all 835 compounds. After excluding 138 compounds out of applicability domain, the coverage was reduced to 83.5%, but the predictive accuracy increased to 80.1%

The y-randomization test was performed as well. For the modeling set with real HTS results, there were 599 models that satisfied the criterion of *q*^2^*/R*^2^ > 0.65 (Table 3), whereas for the data set with randomized HTS results, only 5 models that had *q*^2^*/R*^2^ > 0.65 were generated. These results indicate that our models are statistically robust.

### The utility of the NTP–HTS data for QSAR modeling of rodent carcinogenicity

A total of 314 NTP–HTS compounds are represented in the CPDB. A summary of HTS activity and rodent carcinogenicity of these agents is shown in Table 5. Seventy-seven percent of the compounds classified by NTP–HTS as “active” are also categorized as rodent carcinogens. On the contrary, only 46% of NTP–HTS “inactive” agents are classified by the CPDB as noncarcinogenic in rodents. At the same time, the large fraction of compounds found inactive in HTS assays effectively renders the current assays insufficient in terms of predicting the *in vivo* toxicity.

To further examine whether *in vitro* NTP–HTS data could improve the prediction accuracy for *in vivo* rodent carcinogenicity testing, we applied the hybrid descriptor-based QSAR modeling that utilized both biological (NTP-HTS output) and chemical [MolConnZ (eduSoft LC)] descriptors. First, all 314 compounds were randomly divided into two sets. The modeling set comprised 264 compounds, whereas 50 randomly selected compounds were designated as the external validation set. After calculating chemical descriptors using the MolConnZ software, we combined the NTP–HTS data (a total of seven binary biological descriptors including the active/inactive call for each cell line separately and one for the entire experiment, i.e., a compound was considered active if it was active in at least one cell line) with the MolConnZ chemical descriptors to create a hybrid chemicobiological descriptor set. Although we appreciate that the six cell lines originate from different organs, it is noteworthy that great similarity was observed in cytotoxicity profiles across the entire panel of cell lines (R. Tice, personal communication). Furthermore, the number of active compounds for each individual cell line is relatively small, thus we combined the data. After using the sphere exclusion method to generate training/test set pairs from the same modeling set of compounds, two types of *k*NN QSAR models were developed. One was built using only the MolConnZ chemical descriptor set (340 variables), and the other was built using the combined chemico-biological descriptor set (347 variables).

**Figure 1:**
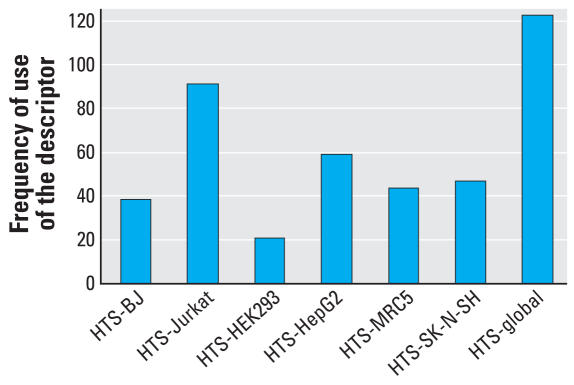
Seven HTS descriptors with their frequency of use in the 198 *k*NN QSAR model.

*k*NN QSAR models were selected based on the *q*^2^*/R*^2^ cutoff of 0.65/0.65 in this modeling development process. One hundred three *k*NN models developed using chemical descriptors alone that passed these criteria, whereas this number nearly doubled to 198 when a combined chemico-biological descriptor set was used. Although data from each of the six cell lines or their combination were given equal weight in defining the global NTP–HTS activity of each compound, the prognostic value of each cell line varied with regard to its usefulness for predicting the rodent carcinogenicity of a chemical. Figure 1 shows the frequency of use of each biological descriptor in the 198 successful *k*NN QSAR models. The predictive power of the QSAR models was verified using the external validation set of 50 compounds not used in training set modeling (Table 6). QSAR modeling using MolConnZ descriptors only [referred to as *k*NN-MolConnZ (*k*NN-MZ) models] achieved 69.2% sensitivity and 55.5% specificity (Table 7). In contrast, 78.6% sensitivity and 66.7% specificity were achieved when the combined chemicobiological descriptor set (referred to as *k*NN-MZHTS models) was used for modeling. The overall prediction accuracy rate increased significantly from 62.3% to 72.7% and the coverage of the external set increased from 88% to 92%, that is, more external compounds were found within (numerically) the same applicability domain when using the hybrid descriptor set.

The y-randomization test was also performed for the carcinogenicity modeling using MZ descriptors only and using the MZ and HTS descriptors. Using randomized carcinogenicity results, no models could be found to satisfy the criterion of *q*^2^*/R*^2^ > 0.65, indicating that our models were statistically robust.

## Discussion

This study evaluated the potential of HTS cell assays as novel biological predictors of adverse health effects caused by chemicals *in vivo* in animal studies. To this end, we have evaluated the HTS data for hundreds of chemical agents tested in six cell lines and focused on compounds that were also studied for their carcinogenicity in chronic two-year cancer bioassays by the NTP. Although HTS results provided complete dose–response data, we used only binary activity summary data (i.e., actives or inactives) because of the binary nature of the CPDB data (i.e., carcinogenic or not carcinogenic). Our initial analysis has established a strong correlation between the chemical structures of the compounds and their effects in cell-based assays. However, we have demonstrated, not surprisingly, that the results of testing compounds in cell viability assays do not serve as unequivocal predictors of their carcinogenicity *in vivo*. Specifically, the data indicated a fairly strong predictivity of cell growth inhibition toward animal carcinogenicity (i.e., a positive cell viability assay response has a strong probability of predicting carcinogenicity *in vivo*) but low, if any, predictivity of the *in vivo* carcinogenicity on the compound effects in cell viability assays (i.e., there are many carcinogens that do not elicit responses in the cell viability assays). Thus, to maximize the utility of *in vitro* assays results for predicting the *in vivo* data, we considered building QSAR models of the *in vivo* chemical carcinogenicity using HTS results as additional biological descriptors of underlying chemical structures.

There are several major potential applications of biological descriptors in QSAR modeling that may advance the science and practice of computational toxicology. In our computational experiments, the binary contributions of all six HTS cell line test results were treated equally *a priori*. The variable selection *k*NN QSAR approach yielded 198 externally predictive models. Because of the nature of the method, these models differ in the choice of descriptors resulting from the variable selection procedure for the final model. Thus, the models could be analyzed for the frequency of occurrence of different descriptors that could reveal chemical determinants of a compound’s carcinogenicity, as well as possible utility of the individual HTS assays. Figure 1 shows the frequency of occurrence of seven HTS descriptors in the 198 *k*NN QSAR models described above. The analysis of this distribution, especially in the context of chemical structure of tested compounds, may provide clues concerning the usefulness of different cell lines for screening purposes.

For example, the HTS–Jurkat and HTS–HepG2 biological descriptors were found in the majority of the successful models. Jurkat and HepG2 are human tumor cell lines derived from a T-cell leukemia and hepatocellular carcinoma, respectively. Jurkat cells grow in suspension with a relatively fast doubling time of about 22 hr. In contrast, HepG2 cells grow as attached cultures with a doubling time of about 37 hr. Both cell lines retain some metabolic capacity toward xenobiotics and are used frequently for *in vitro* testing (Mersch-Sundermann et al. 2004; Nagai et al. 2002). Compared with HTS–Jurkat and HTS–HepG2 cells, the HTS–HEK293 descriptor (a human embryonic kidney cell line) was found in much smaller numbers of successful models, and all but two compounds active in this cell line were also found to be active in other cell lines. Therefore, assay results for the tested compounds in HEK293 cells may be redundant with respect to rodent carcinogenicity modeling conducted here.

Interestingly, the predictions for 8 of the 50 compounds in the external test set were different using the *k*NN-MZ versus *k*NN-MZHTS models. The apparent reason for this disparity (in the context of *k*NN QSAR approach used in this study) is due to the change of nearest neighbors in the training set of these 8 compounds using the MolConnZ (eduSoft LC) descriptors only versus using the hybrid chemical–HTS descriptors. For example, the compound 2,4-dichlorophenol (CAS no. 120-83-2) has 1,2-benzenediol (CAS no. 120-80-9), 1,4-benzenediol (CAS no. 123-31-9), and 4-chlorobenzene-1,2-diamine (CAS no. 95-83-0) as its nearest neighbors in the training set as defined by *k*NN-MZ modeling (Table 6). After including HTS descriptors, its nearest neighbors in the training set change to 2-chloro-*p*-phenylenediamine (CAS no. 61702-44-1), 1-amino-4-methoxybenzene (CAS no. 20265-97-8), and *p*-nitroaniline (CAS no. 100-01-6) instead. Thus, the addition of HTS descriptors affects the similarity relationships between compounds based purely on their chemical descriptors. As shown in this study, the addition of HTS descriptors, on average, improves the prediction accuracy of *in vivo* carcinogenicity.

We further analyzed the interplay between the significance of the bioassay and that of specific chemical descriptors in the context of *in vivo* carcinogenicity by comparing the occurrence of top chemical descriptors in QSAR models with and without HTS descriptors. Table 8 shows chemical descriptors that occur most frequently in successful (i.e., externally predictive) QSAR models using chemical descriptors only. This table also reports the change in occurrence of these descriptors after HTS descriptors are included. Because the number of successful *k*NN QSAR models increased significantly from 103 to 198 after HTS descriptors were used, we also include in Table 8 the ratio of occurrence to the total number of models, which may better indicate the significance of the descriptors.

The descriptors for each final *k*NN QSAR model are chosen as a result of the stochastic variable selection procedure that maximizes the correlation between descriptors and carcinogenicity. We reasoned that the analysis of occurrence of various chemical descriptors before and after inclusion of HTS descriptors in modeling may be interpreted in terms of their relative information content with respect to the *in vivo* toxicity. Thus, those chemical descriptors that have a similar ratio of occurrence in models with or without HTS descriptors (exemplified by descriptors 1, 2, and 7) contribute to successful models independently of the biological descriptors. For compounds whose predicted activity is primarily determined by the presence of these particular chemical descriptors and unaffected by the addition of HTS descriptors, this implies that the HTS adds no new information to the prediction of *in vivo* carcinogenicity. Conversely, if the frequency of a chemical descriptor decreases significantly after the HTS descriptors are included, it is less important than, and likely redundant with, the biological descriptors. In these cases, the biological descriptor is clearly adding new, biologically significant information that is not as effectively captured by the chemical descriptor.

Interestingly, descriptors 1 and 2 (*N*-nitroso compounds) were selected as the most important chemical descriptors in our models, and their importance is relatively unaffected by inclusion of HTS descriptors. The large majority of *N*-nitroso compounds have been found to produce genotoxic effects and to cause tumor development in laboratory animals, as they are metabolized to reactive electrophilic species causing damage to various cellular constituents such as DNA, constituting a key event in the carcinogenic mechanism (Brambilla and Martelli 2007). Because such metabolic transformations do not generally occur in cellular systems, the significance of all but one of the HTS assays (i.e., HepG2) in predicting events relevant to the carcinogenicity for these compounds is likely to be minimal. To the contrary, the NTP–HTS data show that cells are highly sensitive to the effects of amine-type compounds (descriptors 6 and 10) and biological descriptors are better predictors of the carcinogenic potential of these agents than structure alone. Among all 30 carcinogens that are also active in HTS tests, 15 are amines. A similar observation can be made for organic compounds containing phosphorus (descriptor 8). Most of the remaining chemical descriptors, which approximately delineate neighborhoods of chemical space, have similar distribution among the models with or without the HTS descriptors. Hence, HTS descriptors offer no additional value as predictors of carcinogenicity for these chemical subsets. As more HTS data are generated, the above analysis suggests a strategy that can be used to elucidate possible mechanistic relevance of HTS assays to carcinogenicity prediction within areas of chemical space approximately defined by chemical descriptors.

## Conclusions

We have examined the utility of *in vitro* NTP–HTS data for predicting *in vivo* adverse health effects (i.e., carcinogenicity) of environmental agents. Our analysis suggests that NTP–HTS results have limited predictive power by themselves for rodent carcinogenicity. This result is not surprising, given the relatively low frequency of positives across the HTS assays (16%) and that cell viability (i.e., cell death) may not be directly related to the carcinogenic potential of a compound. However, our data suggest that using the NTP–HTS results as biological fingerprint descriptors of generalized xenobiotic-induced pathophysiological processes helps improve the overall QSAR-based prediction accuracy of rodent carcinogenicity compared with those based on chemical descriptors alone. While the mechanistic relevance of the HTS assays in predicting rodent carcinogenicity is unclear at present, the empirical evidence of the significance of the biological descriptors for the computational modeling purposes is compelling and should motivate continued investigation. Furthermore, as additional sets of compounds with known *in vivo* toxicity responses are investigated in cell-based viability assays, we shall continue to develop models similar to those reported in this article for additional toxicity end points. The present analysis suggests that as more mechanistically relevant HTS data are generated and a greater number of compounds are screened, computational toxicology tools could be used to select most relevant HTS assays (cell lines and/or measurements) and prioritize chemical agents for screening. With sufficient improvements in resulting model predictive performance, *in vitro* HTS bioassays, coupled with traditional chemical structure-based descriptors, may be ultimately helpful in prioritizing or even partially replacing *in vivo* toxicity testing.

## Correction

The following corrections have been made from the original manuscript published online. In the Abstract under “Methods and Results,” the phrase “curated data set of 557 compounds” has been changed to “curated data set of 384 compounds.” The sentence “The resulting models had prediction accuracies for training, test (containing 400 compounds together), and external validation (157 compounds) sets as high as 79%, 79%, and 84%, respectively” has been changed to “The resulting models had prediction accuracies for training, test (containing 275 compounds together), and external validation (109 compounds) sets as high as 89%, 71%, and 74%, respectively.”

## Figures and Tables

**Table 1:** Summary of the biological activity of chemical agents screened in NTP–HTS assays.

Classification	BJ	HEK293	HepG2	Jurkat	MRC-5	SK-N-SH	All tests
Actives	42	63	41	121	37	74	140
Inconclusives	44	79	47	89	44	54	90
Inactives	1,203	1,147	1,201	1,079	1,208	1,161	1,059

**Table 2:** Rodent carcinogenicity classification (CPDB database) for 314 NTP–HTS compounds.

	Rats		Mice	
Classification	Male	Female	Male	Female
Active	121	111	123	134
Inactive	150	154	153	140
Total	271	265	276	274

**Table 3:** Statistical information of the 15 most statistically significant *k*NN QSAR models based on the 275-compound modeling set.

Model ID	N-training	Pred.-training	N-test	Pred.-test	NNN
1	141	0.90	119	0.73	1
2	140	0.91	121	0.71	1
3	141	0.92	120	0.69	1
4	140	0.88	123	0.73	1
5	141	0.88	120	0.73	1
6	190	0.90	85	0.71	1
7	228	0.86	47	0.74	1
8	140	0.92	121	0.67	1
9	140	0.89	116	0.70	4
10	149	0.88	122	0.70	1
11	140	0.87	124	0.72	1
12	190	0.85	85	0.73	1
13	140	0.88	125	0.70	1
14	149	0.87	118	0.71	1
15	141	0.92	123	0.66	1
Average	154	0.89	111	0.71	1

Abbreviations: N-training, number of compounds in the training set; Pred.-training, the overall predictivity of the training set; N-test, number of compounds in the test set; Pred.-test, the overall predictivity of the test set; NNN, number of the nearest neighbors used for prediction.

**Table 4:** Consensus prediction for 109 compounds in the external validation set.

	Consensus prediction		After applicability domain applied	
Model characteristics	Exp. actives	Exp. inactives	Exp. actives	Exp. inactives
Pred. actives (*n*)	21	7	17	5
Pred. inactives (*n*)	16	65	9	65
Sensitivity (%)	56.8		65.4	
Specificity (%)	90.2		92.9	
Overall predictive power (%)^a^	73.5		79.2	

Abbreviations: Exp., experimental; Pred., predicted.

a The overall predictive power is the average value of sensitivity (predictive rate of actives) and specificity (predictive rate of inactives).

**Table 5:** The relationship between HTS activity and rodent carcinogenicity of 314 compounds.

Content of CPDB	HTS actives	HTS inconclusives	HTS inactives
CPDB actives (*n*)	30	12	136
CPDB inactives (*n*)	9	13	114
Correlation (%)	77	—	46

**Table 6:** Consensus prediction of 50 compounds in the external validation set using the *k*NN QSAR models based on two different descriptor sets.

CAS no.	Name	CPDB actives	MZ	MZHTS
79005	1,1,2-Trichloroethane	+	+	+
106934	1,2-Dibromoethane	+	+	+
90120	1-Methylnaphthalene	–	–	–
86577	1-Nitronaphthalene	–	+	+
634935	2,4,6-Trichloroaniline	+	+	+
120832	2,4-Dichlorophenol	–	+	–
99558	5-Nitro-*o*-toluidine	+	+	+
67630	Isopropanol	–	+	–
96695	4,4-Thiobis(6-*tert*-butyl-*m*-cresol)	–	+	+
619170	4-Nitroanthranilic acid	–	–	–
298817	8-Methoxypsoralen	+	+	+
75058	Acetonitrile	–	+	+
50782	Acetylsalicylic acid	–	–	–
50760	Actinomycin D	+	I	+
86500	Azinphosmethyl	–	–	–
92875	Benzidine	+	+	+
57578	Propiolactone	+	+	+
80057	Bisphenol A	–	+	–
75274	Bromodichloromethane	+	+	+
115286	Chlorendic acid	+	I	I
91645	Coumarin	+	+	+
4342034	Dacarbazine	+	–	–
103231	Di(2-ethylhexyl)adipate	+	+	+
333415	Diazinon	–	–	–
62737	Dichlorvos	+	+	+
828002	Dimethoxane	+	–	–
98011	Furfural	+	+	+
87683	Hexachloro-1,3-butadiene	+	–	+
67721	Hexachloroethane	+	+	+
122667	Hydrazobenzene	+	–	–
58935	Hydrochlorothiazide	–	I	I
121755	Malathion	–	–	–
298000	Methyl parathion	–	–	–
150685	Monuron	+	–	–
1212299	*N,N’*-Dicyclohexylthiourea	–	I	I
759739	*N*-Ethyl-*n*-nitrosourea	+	+	+
98953	Nitrobenzene	+	+	+
67209	Nitrofurantoin	+	I	+
59870	Nitrofurazone	+	I	I
55185	*N*-Nitrosodiethylamine	+	+	+
636215	*o*-Toluidine hydrochloride	+	–	–
106478	*p*-Chloroaniline	–	–	–
122601	Phenyl glycidyl ether	+	+	+
103855	Phenylthiourea	–	–	+
1918021	Picloram	–	–	–
57681	Sulfamethazine	+	–	–
79196	Thiosemicarbazide	–	+	+
108054	Vinyl acetate	+	+	+
1330207	Xylenes (mixed)	–	+	+
17924924	Zearalenone	+	–	+

Abbreviations: +, carcinogenic; –, noncarcinogenic; I, inconclusive because out of the applicability domain; MZ, models based on MolConnZ descriptors only; MZHTS, models based on the combination of MolConnZ and HTS descriptors.

**Table 7:** Summary of the statistical parameters of the prediction results of 50 external compounds.

	Chemical descriptors only		Combined descriptors	
Model characteristics	Exp. actives	Exp. inactives	Exp. actives	Exp. inactives
Pred. actives	18	8	22	6
Pred. inactives	8	10	6	12
Sensitivity (%)	69.2		78.6	
Specificity (%)	55.5		66.7	
Overall predictivity (%)	62.3		72.7	
Coverage (%)	88		92	

Abbreviations: Exp., experimental; Pred., predicted.

**Table 8:** Summary of the top 10 atom and bond type MozConnZ chemical descriptors used in successful *k*NN QSAR models before and after using HTS descriptors.

No.	Descr_Name	Illustration	Freq_MZ	Ratio_MZ	Freq_MZHTS	Ratio_MZHTS
1	Snitroso	Sum of E-states of nitroso group 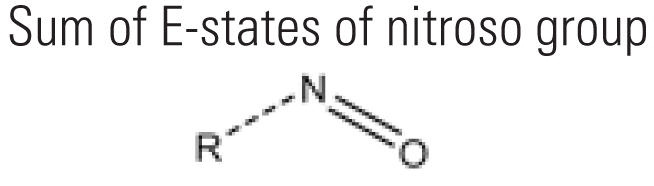	38	36.9%	73	36.9%
2	nnitroso	Number of nitroso group 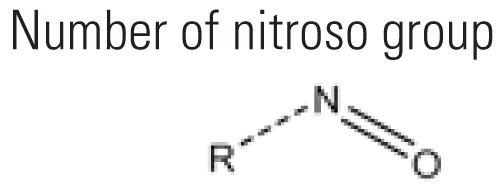	34	33.0%	69	34.8%
3	nHBint3	Number of hydrogen bond acceptor and donor pairs separated by 3 skeletal bonds 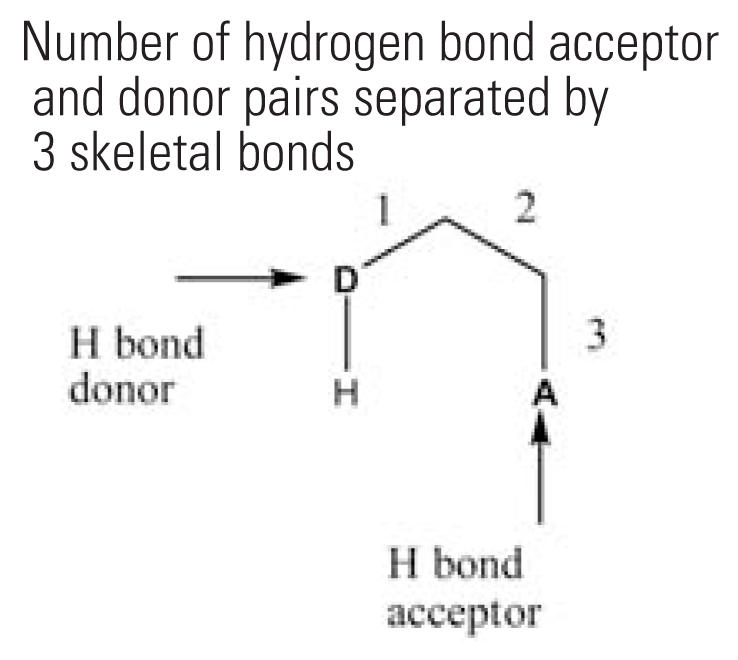	27	26.2%	31	15.7%
4	naasN	Number of aromatic nitrogen with substitute 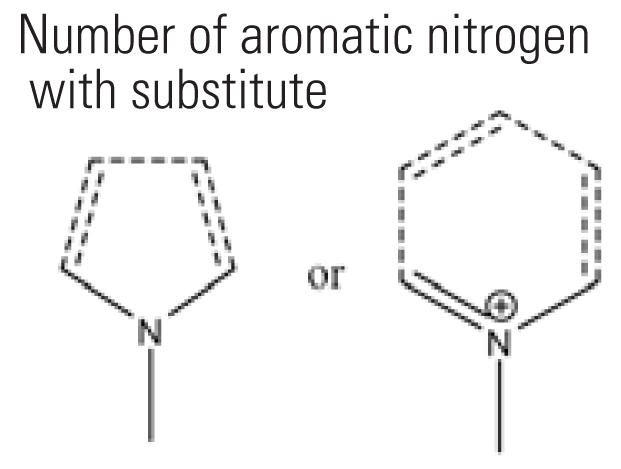	25	24.3%	42	21.2%
5	SHBint3	Sum of E-state of strength for potential hydrogen bonds if separated by 3 skeletal bonds 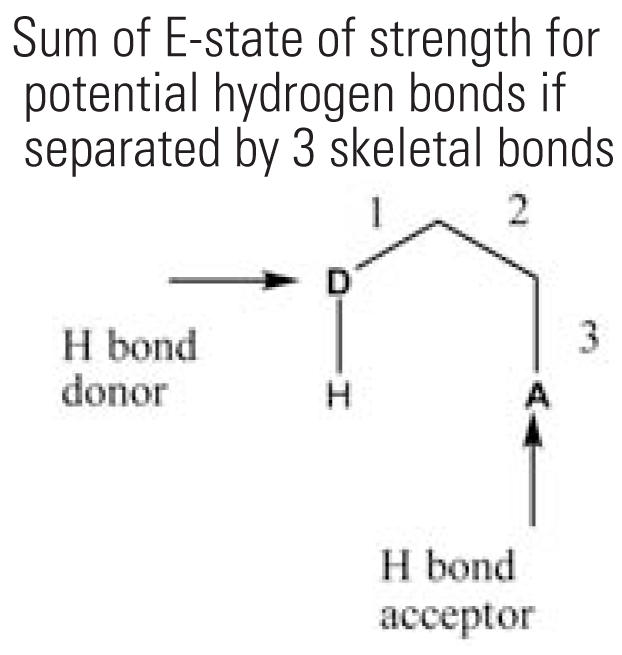	24	23.3%	41	20.7%
6	nHssNH	Number of amine groups 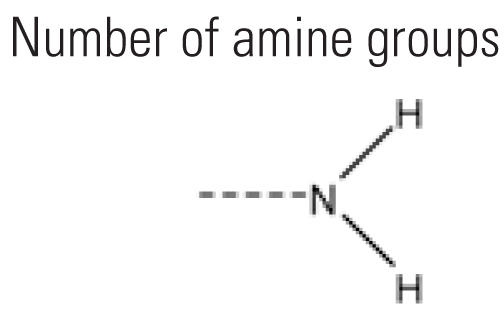	24	23.3%	23	11.6%
7	SdsN	Sum of E-states for nitrogens with one single bond and one double bond 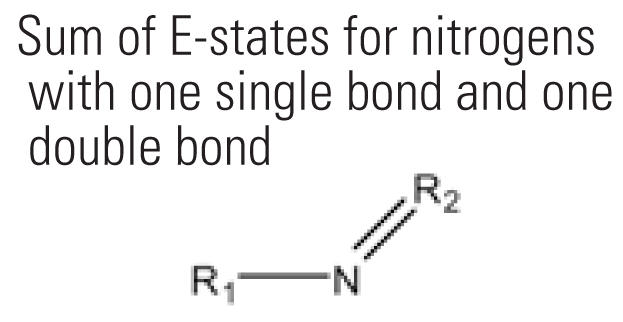	24	23.3%	48	24.2%
8	SdsssP	Sum of E-states for phosphors with three single bonds and one double bond 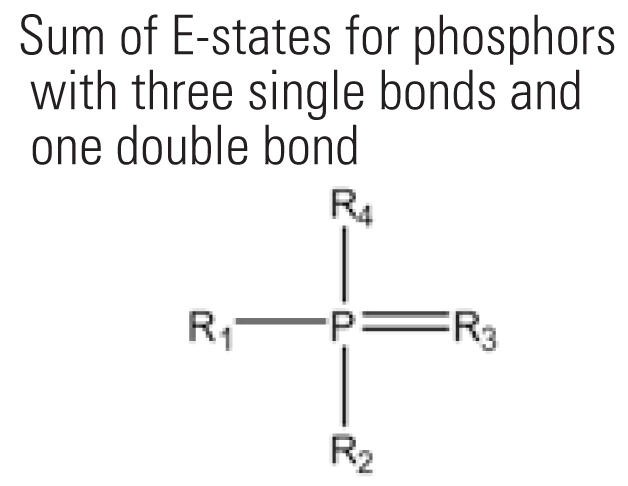	19	18.4%	21	10.6%
9	SsBr	Sum of E-states for bromines 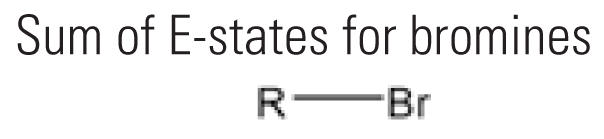	19	18.4%	45	22.7%
10	SHssNH	Sum of H E-states for hydrogens in amine groups. 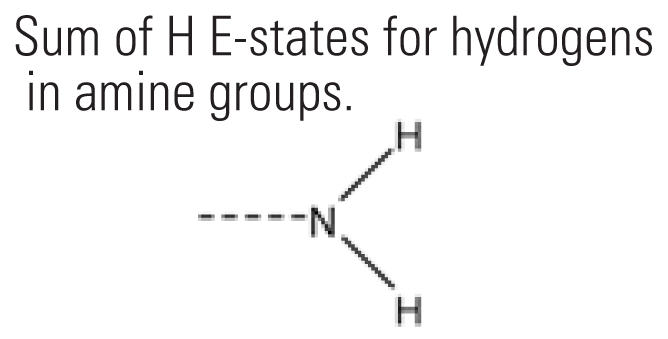	18	17.5%	25	12.6%

Abbreviations: Descr_Name, name of descriptor; Freq_MZ, frequency of occurrence in successful *k*NN models only using only MolConnZ descriptors; Ratio_MZ, ratio of occurrence in successful QSAR models using only MolConnZ descriptors; Freq_MZHTS, frequency of occurrence in successful *k*NN models using MolConnZ and HTS descriptors; Ratio_MZHTS, ratio of occurrence in successful QSAR models using MolConnZ and HTS descriptors.
